# Comparative Effectiveness of Physical Activity Intervention Programs on Motor Skills in Children and Adolescents: A Systematic Review and Network Meta-Analysis

**DOI:** 10.3390/ijerph191911914

**Published:** 2022-09-21

**Authors:** Mohamed A. Hassan, Wenxi Liu, Daniel J. McDonough, Xiwen Su, Zan Gao

**Affiliations:** 1 School of Kinesiology, University of Minnesota-Twin Cities, 208 Cooke Hall, 1900 University Ave. SE, Minneapolis, MN 55455, USA; 2 Department of Methods and Curriculum, Physical Education College for Men, Helwan University, Cairo 12552, Egypt; 3 Department of Physical Education, Shanghai Jiao Tong University, Shanghai 200240, China; 4 School of Public Health, University of Minnesota-Twin Cities, Minneapolis, MN 55455, USA; 5 School of Rehabilitation Science, Boston University, Boston, MA 02215, USA

**Keywords:** fundamental motor skills, object control, locomotor, gross motor, exergaming, physical activity

## Abstract

**Objective**: To evaluate how different physical activity (PA) interventions (traditional, exergaming, and teacher/parent education) impacted children’s motor skills (object control, locomotor, and gross motor). **Design**: Systematic review and network meta-analysis. **Data sources**: PubMed, Medline, Scopus, Web of Science, EMBASE, and PsycINFO. **Eligibility criteria**: (1) Participants comprised 1708 children 3–12 years; (2) PA or exercise-based interventions were investigated; (3) only studies using a Test of Gross Motor Skills assessment were included; (4) RCT were chosen as the study design to assess the impact of PA interventions on children’s motor skills; and (5) culture-based PA studies with English language only were included. Data were analyzed using a Bayesian network meta-analysis. **Results**: The results were reported as standardized mean differences (SMDs) with associated 95% credible intervals (CrIs). For object control, aerobic intervention (SMD 6.90, 95% Crl 1.39 to 13.50); for locomotor, exergaming intervention (SMD 12.50, 95% Crl 0.28 to 24.50); and for gross motor, aerobic intervention (SMD 7.49, 95% Crl 0.11 to 15.70) were the most effective treatments. **Conclusion**: Children’s FMSs have been improved through different PA interventions. Among them, aerobic interventions seem to be the most effective intervention in enhancing object control skills and overall gross motor skills.

## 1. Introduction

The Centers for Disease Control and Prevention (CDC) released a report indicating the high increase in childhood obesity in the U.S. [1,2]. It is estimated that the prevalence of obesity has affected about 14.7 million children and adolescents. With that being said, it is also worth noting that, due to the COVID-19 pandemic outbreak [3], scientists expected the risk of obesity to be increased, especially during school closures and the sedentary behavior prevalence [4,5]. While the need to halt the rise of risks became essential, being engaged in physical activity (PA) settings appeared to play an imperative role in maintaining a healthier lifestyle to curb childhood obesity [6,7]. Moreover, studies targeting school-based PA interventions have proven the positive influence of school programs on promoting PA and decreasing obesity among children [8,9]. However, many researchers in the current literature have investigated different PA measurements and programs. Focusing on learning how to move, known as fundamental movement skills (FMSs), remains notably underestimated [10].

Recent studies have adopted different approaches to promote PA levels among children by linking PA and targeted physical benchmark goals in school settings [11]. Schools tend to be the most appropriate learning environment to learn basic movement skills at a young age, given the fact that children usually spend more than 50% of their time awake at school [12]. In essence, the focus on enhancing FMSs is to establish a smooth transition from different fine motor skills to gross motor skills, including locomotor skills (e.g., walking, running, and jumping), object control skills (e.g., catching, throwing, kicking), and body control skills (e.g., balancing, swinging) [13]. Accordingly, many researchers examined a variety of PA interventions, such as active video games (AVG), on youth’s FMSs in the past decade [14].

As known, children’s FMSs can be enhanced through different PA intervention programs, including and not limited to equity-based active play interventions [15], object control skills [16], gender-specific PA programs [17], school-based PA programs [18], and technology-based gaming programs [19]. In addition, several randomized controlled trials (RCTs) have demonstrated the importance of using exergaming (e.g., Xbox, Nintendo Wii) in enhancing locomotor skills [20] or FMSs [21]. Therefore, researchers tend to examine the impact of exergaming on FMS improvement as it is recognized as a new technological tool that could be implemented in school-based programs. Moreover, a series of recent studies indicated the objectivity of employing major teaching techniques in PA; results refer to the influence of teacher support to promote and develop FMSs [22,23]. As a consequence, while the recent studies’ empirical outcomes have varied due to each specific PA intervention, it has become essential to conduct a study to determine and assess whether there is a superiority of a specific PA intervention over the others.

Thus far, a number of narrative systematic reviews and meta-analyses have described the effects of PA on promoting motor skills among children [24,25,26,27,28,29]. However, it is still not clear which PA interventions led to which subcategories of FMSs among pediatric populations. Therefore, the purpose of this review was to systematically evaluate how different PA interventions impacted children’s motor skills by using an advanced statistical technique — network meta-analysis. The findings of this review might be beneficial to scholars, educators, and health professionals to better understand the underlying mechanisms of PA interventions that enhance children’s motor skills with the goal of helping them develop and maintain a physically active lifestyle.

## 2. Methods

In this review, we adopted the Preferred Reporting Items for Systematic Review and Meta-Analysis Protocols (PRISMA-P) statement for reporting [30].

### 2.1. Information Sources and Search Strategies

Three investigators (WL, XS, and ZG) conducted the screening and selection process independently before May 2022. The peer-reviewed articles were searched via different databases that included PubMed, Medline, Scopus, Web of Science, EMBASE, and PsycINFO. The investigators used the following terms and keywords in different combinations during the literature search process: (“Physical Activity” OR “Physical Exercise” OR “Exergaming” OR “Active Video Games” OR “Aerobic Exercise”) AND (“Motor Skills” OR “Fundamental Motor Skills” OR “Motor Skill Competence” OR “Object Control Skills” OR “Locomotor Skills” OR “Gross Motor Skills” OR “Motor Development”) AND (“School Children” OR “Pre-School Children”).

### 2.2. Eligibility Criteria

Eligibility criteria were formulated by using the population, intervention, comparisons, outcomes, and study (PICOS) framework [30]. In detail, the following inclusion elements were used for each study: (1) participants comprised preschool children (age 3 to 5.9 years) and school-aged children/adolescents (age 6 to 12 years). Notably, disabilities were not an exclusion criterium, and thus a few studies included children with ADHD [31] or DCD [32]; (2) PA or exercise-based interventions were investigated and assessed; (3) only studies with quantitative measures retrieved from using Test of Gross Motor Skills (TGMD) versions (1, 2, and 3) [33,34,35] as an assessment tool to examine differences noted before, during, or after treatments were included; (4) RCT was chosen as the study design to assess the impact of PA interventions on children’s motor skills; and (5) several culture-based PA approaches from different countries were employed, but only English language studies [36] were included in this review.

### 2.3. Comparators

Children’s FMSs included three subgroups: object control skills, locomotor skills, and gross motor skills. PA Interventions with two or more treatment arms were employed. The authors have coded four comparators as: (1) control group (e.g., usual care, waiting list); (2) aerobic group (e.g., traditional PA interventions); (3) exergaming (e.g., Xbox, Nintendo Wii); and (4) parent/teacher education interventions.

### 2.4. Data Extraction and Processing

In the first stage of the literature search, the titles and abstracts of identified articles were checked for relevance by three investigators (WL, XS, and ZG). In the second stage, the full-text articles were retrieved and considered for review based on the inclusion criteria. All relevant articles were stored in a shared online Google folder, and all authors were able to access the folder for reviewing and editing the content. In the final stage, two investigators (WL and XS) went through the included articles to extract FMSs-related data (e.g., mean differences and standard deviations), and they were checked for accuracy by the third author (ZG).

### 2.5. Network Geometry

To have a wider visualization of the NMA’s available evidence, we used a conventional network graph containing “nodes”, which represent different treatment options, and “edges”, which are lines between nodes that represent available direct comparisons between those treatments [30]. We used network plots for all three outcomes (locomotor skills, object control skills, and gross motor skills). Notably, node size differs proportionally according to the number of direct comparisons including that treatment node. In detail, a large node represents the treatment that is frequently used as a comparator in the studies. In addition, the thickness of the edges is proportional to the number of studies that compared those direct treatments.

### 2.6. Statistical Analysis

Following the basic NMA structure in the literature review [37,38], the authors made sure to meet all NMA assumptions. For transitivity, it is known that when covariates of effect modifiers are not evenly distributed among the selected interventions in an NMA [37], the possibility of violating the transitivity is high. However, transitivity cannot be statistically evaluated, the authors discussed the possibility of violating this assumption among the selected studies. As aresult, the authors have agreed to include certain age ranges and children’s ability to engage in PA as described in the inclusion criteria, and thus, we have attenuated the risk of violating the assumption of transitivity.

Second, to assess the heterogeneity between PA interventions, we used Bayesian random-effects evidence synthesis models with vague N (0, 1000) prior to assessing the homogeneity of the effects between comparators [39,40]. Further, we have visually inspected the contrast plots for locomotor skills, object control skills, and gross motor skills. In detail, we used the “pcnetmeta” package in R statistical software (Version 1.2.5042, R Foundation) to conduct an arm-based NMA using Bayesian hierarchical modeling derived from Markov Chain Monte Carlo methods [41]. Instead of reporting the significance level of the results, we avoided dichotomization by presenting the results with credible intervals (CrIs) to allow readers to interpret the range of the likelihood of effects [42]. Specifically, comparative standardized mean differences (SMDs) were reported with their associated 95% CrIs with 2.5% and 97.5% quantiles as the lower and upper bounds.

Third, due to the lack of closed loops within any of the three networks (i.e., locomotor skills, object control skills, and gross motor skills), we were unable to assess the consistency [37]. However, we used the Bayesian NMA model to assess heterogeneity, which is considered an inconsistency measure, in addition to validating the assumption of transitivity. Therefore, all authors reached the consensus that consistency would not be violated within our networks. Further, the Cochrane Risk of Bias Assessment Tool for RCTs [43] (Table 1) was used to assess the risk of bias within individual studies.

## 3. Results

### 3.1. Search Results and Study Characteristics

**Figure 1:**
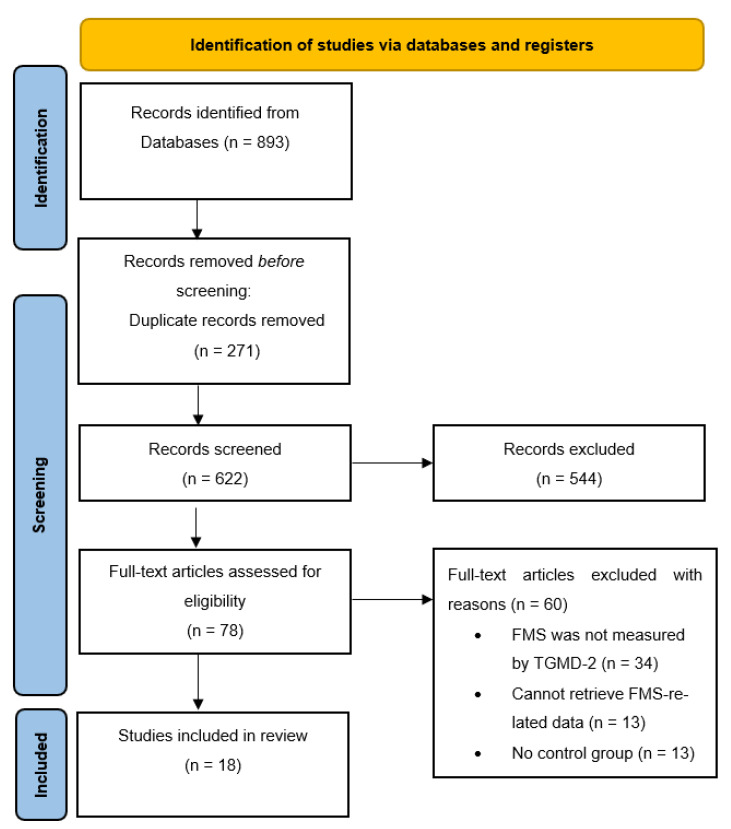
PRISMA flow chart for systematic reviews.

Of 893 studies, 18 (2%) met the inclusion criteria (Figure 1), with an overall sample of 1708 participants divided into two groups: 875 (51%) intervention participants and 833 (49%) control participants. Among them, 11 (61%) studies targeted school-age children [14,15,16,20,21,22,23,31,32,44,46] while 7 (39%) studies targeted preschool-age children [17,18,19,47,48,49,50]. Notably, the sample of recruited school children represented 989 participants out of 1708 (58%), while the sample of recruited preschool children represented the rest of the children (42%).

This NMA included studies examining FMSs; specifically, studies examining object control comprised 16 out of 18 studies [14,15,16,17,18,19,21,22,23,31,32,44,46,47,49,50], while locomotor skills were assessed through 11 out of 18 studies [15,17,19,20,22,23,31,32,44,46,47], and gross motor skills were examined in 13 out of 18 studies [15,16,17,18,19,22,23,31,32,44,46,47,48].

The selected studies were published between 2002 and 2022. Among them, 13 (72%) were published after 2015 [14,15,16,19,20,21,22,23,31,32,47,48,49]. Although only studies published in English were retrieved, surprisingly, the studies represented 9 different countries and regions: the USA [22,47,48,49,50], Australia [14,16,18,44], Greece [21,46], Hong Kong [23,32], Ireland [20], Scotland [15], China [31], Belgium [17], and the UK [19]. Thus, the included studies seemed to indicate an acceptable variation according to cultural differences. Notably, studies from both the USA and Australia [14,16,18,22,44,47,48,49,50] accounted for 50% of all studies.

The intervention duration among all studies ranged from 5 to 52 weeks, with 10 (55%) ranging from 8 to 12 weeks [15,17,20,21,31,46,48,49,50,51]. In addition, 13 (72%) of the studies employed traditional PA or aerobic [15,17,18,19,21,22,23,31,32,44,46,47,50] interventions, while 5 (28%) of the studies used exergaming [14,16,20,21,48] in the PA treatments. Only two studies used parent/teacher education interventions [44,49].

While all studies reported pre- and post-intervention measures using TGMD, only three (17%) of the studies reported mid-point or retention measures [20,32,50], nevertheless, All authors agreed to report only pre- and post-intervention measures in this review. Lastly, only two (11%) studies recruited samples from the children with disabilities population: ADHD [31] and DCD [32].

The overall risk of bias was rated as high for four (22%) studies [16,27,41,45]. In detail, three studies were rated high risk and one study rated medium (unclear) for random sequence generation; three studies were rated high risk and one study rated medium risk for allocation concealment; and three studies were rated high risk and one study rated medium risk for blinding participants and personnel.

### 3.2. Network Geometry

**Figure 2:**
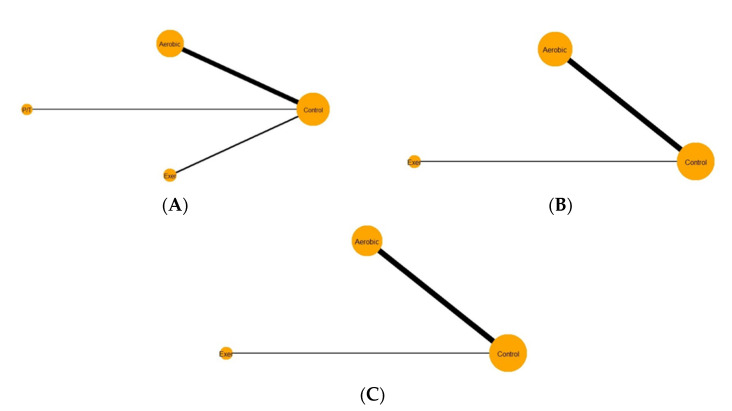
Network plot for object control (**A**), locomotor (**B**), and gross motor (**C**). Abbreviations: Exer = exergaming; P/T = parent/teacher education.

The network plot in object control skills is presented in Figure 2A. In this network, four different comparators were examined. No closed loops were detected among all comparators. In addition, node sizes of control and aerobic comparators were large and similar in size relative to the exergaming and parent/teacher education comparator. Lastly, the edge between control and aerobic comparators is considered the thickest among the other network edges, indicating that this comparison was the most examined within this network.

The network plot in locomotor skills is shown in Figure 2B. In this network, only three different comparators (i.e., control, aerobic, and exergaming) were examined, while the teacher/parent education comparator was excluded due to limited evidence of direct treatment. Similarly, no closed loop among all comparators was identified. In addition, node sizes of control and aerobic comparators were similar in size and large relative to exergaming. Edge thickness was greatest between control and aerobic comparators, indicating a large number of direct comparisons between those nodes.

The network plot in gross motor skills is shown in Figure 2C. Similarly, only three different comparators were examined (i.e., control, aerobic, and exergaming). No closed loops were observed, either. The node sizes for control and aerobic comparators were large relative to exergaming. Moreover, a thick edge was noticed between control and aerobic comparators compared to that of exergaming intervention.

Apparently, looking at the three network plots, we noticed that the largest number of treatment comparisons were concentrated on the head-to-head comparison between control and aerobic comparators, while exergaming treatments were well reflected in the three networks. In addition, the teacher/parent comparator was observed in one network only (i.e., object control skills).

### 3.3. Network Meta-Analysis

**Figure 3:**
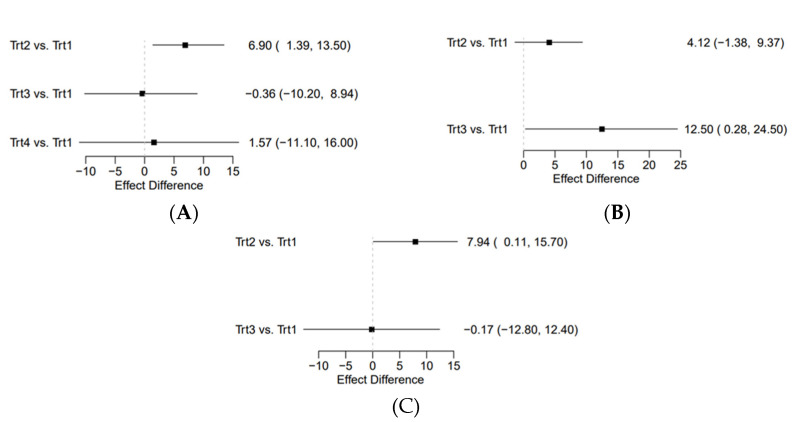
Contrast plot for object control outcome (**A**), locomotor outcome (**B**), and gross motor outcome (**C**). Abbreviations: Trt1, reference intervention arm (control); Trt2, aerobic (traditional intervention); Trt3, exergaming intervention; Trt4, parent/teacher education intervention. Squares represent comparative standardized mean differences, and their associated lines are 95% credible intervals with 2.5% and 97.5% quantiles as the lower and upper bounds.

**Figure 4:**
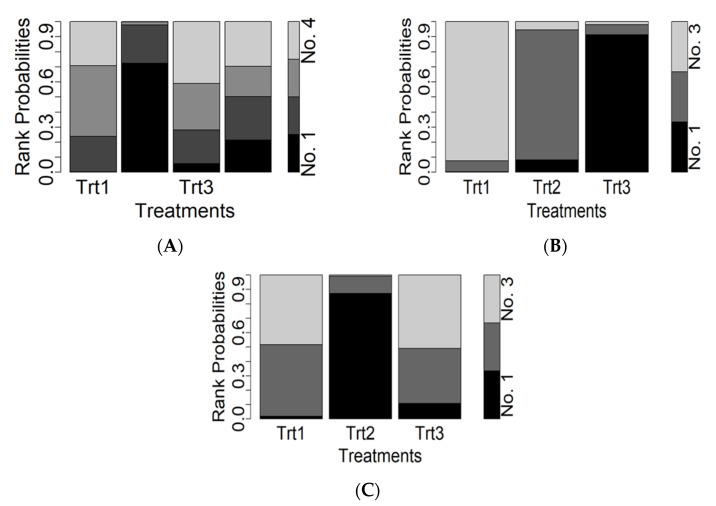
Plots of treatment rank probabilities for object control (**A**), locomotor (**B**), and gross motor (**C**). Trt1, reference intervention arm (control); Trt2, aerobic intervention; Trt3, exergaming intervention; Trt4, parent/teacher intervention. A darker area indicates the probability of a higher rank.

For changes in object control skills, 16 studies compared three different PA intervention approaches (783 children) against controls (732 children). Compared with the control, the children’s objective control skills increased remarkably with aerobic interventions as compared to exergaming and teacher/parent education interventions (Figure 3A). The SMDs for changes in object control skills ranged from −0.36 (95% Crl −10.20 to 8.94) for exergaming interventions to 6.90 (95% Crl 1.39 to 13.50) for aerobic interventions. Notably, the only intervention which did not include 0 in the 95% Crl is the aerobic intervention. Its Crl was the most precise among all comparators, which indicates that aerobic intervention was the best intervention in promoting children’s objective control skills. Ranking based on the degree of FMS development confirmed the aerobic intervention as the most effective and the control condition as the least effective program (Figure 4A).

For changes in locomotor skills, 11 studies compared 2 different PA interventions (503 children) against the control condition (518 children). Compared with the control, children’s locomotor skills increased greatly with exergaming interventions rather than aerobic interventions (Figure 3B). The SMDs for changes in locomotor skills ranged from 4.12 (95% Crl −1.38 to 9.37) for aerobic interventions to 12.50 (95% Crl 0.28 to 24.50) for exergaming interventions. Notably, the aerobic interventions included 0 in the 95% Crl and the Crl was not precise. For exergaming, however, the range of Crl was relatively large, but it did not include 0, which suggests a better outcome compared to the aerobic interventions. Ranking based on the degree of FMS development identified the exergaming intervention as the most effective, whereas the control intervention is the least effective (Figure 4B).

For changes in gross motor skills, 13 studies compared 2 different PA interventions (570 children) against the control condition (539 children). Compared with controls, children’s gross motor skills increased remarkably with aerobic interventions compared to exergaming interventions (Figure 3C). The SMDs for changes in gross motor skills ranged from −0.17 (95% Crl −12.80 to 12.40) for exergaming interventions to 7.49 (95% Crl 0.11 to 15.70) for aerobic interventions. Similar to object control skills, the aerobic interventions were more effective in improving gross motor skills than exergaming interventions. Again, ranking based on the degree of FMS development indicates the aerobic intervention as the most effective and the control condition as the least effective program (Figure 4C).

## 4. Discussion

This NMA comprehensively investigated the effects of different PA interventions (i.e., traditional PA or aerobic interventions, exergaming interventions, and teacher/parent education interventions) on children’s FMSs, including object control skills, gross motor skills, and locomotor skills.

In detail, we expected that exergaming might have the greatest positive effects on children’s object control skills [52], as it is the most attractive and fun PA intervention for children at this age. Yet, surprisingly, aerobic interventions emerged as the most effective program as compared to exergaming and other programs. Simultaneously, other studies’ results confirmed our findings. For instance, Ye et al. [53] examined the effectiveness of a combined physical education (PE) and exergaming program on children’s motor competence skills. Their results, specifically, indicated that children in the traditional PE program demonstrated greater improvement in object control skills (kicking and throwing) when compared to the exergaming group. Moreover, Liu et al. [54] systematically reviewed nine randomized controlled trials (RCTs) and one quasi-experimental study to synthesize the most updated literature defining the impact of AVG on FMSs. Their findings suggested that using AVG to improve object control and locomotor skills needed more investigation, as the results revealed no significant impact. In addition, McDonough et al. [55] systematically reviewed 25 RCTs to synthesize the literature regarding the effect of traditional and exergaming-based interventions on motor skill development. However, while their results confirmed the causal relationship between increased PA and motor skills development, findings to support the development of object control skills remain inconclusive. Worth mentioning still, they also noted some limitations in the literature in terms of long-term interventions or retention studies, which might lead to different findings [56].

As for the findings for locomotor skills, most of the recruited studies have investigated the influence of different interventions on locomotor skills. Unlike object control skills, exergaming interventions appeared to be the most effective when compared to other approaches. Aligning with our results, a previous study has shown supporting evidence [53] as motor competence skills were analyzed to evaluate the impact of using an exergaming-based PA program. However, the findings were not significant, but children who used exergaming demonstrated slightly better improvement in skills, such as hopping, standing, and long jump. Another study supported our findings: Comeras-Chueca et al. [57] systematically reviewed 20 articles to synthesize the literature regarding the effect of AVG on motor competence. Their findings illustrated improvements in several motor skills, such as long jump distance, hopping, kicking, and throwing. Moreover, their results agreed with the previous study’s outcome, indicating no significance but a slight improvement in locomotor skills. In addition, Liu et al. [54] reported similar results indicating the impact of AVG on improving several motor skills. Although, they reported that their findings are neither consistent nor conclusive due to the mixed reults reported in the recruited studies in their review, where some studies reported no significant difference, the overall findings illustrated a positive impact of AVG on imporving FMS and physical fitness. 

The findings for gross motor skills aligned with those of PA interventions on object control skills. Specifically, aerobic interventions were reported to be the most effective program for children’s gross motor skills. Moreover, our findings are in line with several previous studies. For instance, Ruiz-Esteban et al. [58] evaluated the impact of motor intervention PE programs on the development of gross motor skills. Their findings were aimed at the improvement of some gross motor skills, such as walking, arm coordination, and leg coordination. Following the same trend, Brusseau et al. [59] examined the influence of using a comprehensive school physical activity on school-day step count, health-related fitness, and gross motor skills. The findings aimed to determine the effectiveness of using the PE program for improving gross motor skills and cardiorespiratory endurance. In addition, Burns et al. [60] examined the effect of using a 12-week PA program on gross motor skills. The results indicated an approximate 10% improvement in gross motor skills from baseline to 12-week testing. Their findings are in line with our study illustrating the effectiveness of traditional or aerobic PA interventions.

With that being said, we can conclude that using the traditional or aerobic interventions demonstrated better FMS outcomes, specifically with object control and gross motor skills rather than exergaming interventions. This positive impact could result from the effectiveness of the regular PE programs implemented in schools and preschools. Moreover, other variables—that were not evaluated in our NMA—might have contributed to the positive effects of traditional or aerobic interventions, such as psychological influences resulting from playing with peers or in groups and physical activity competency. In addition, intervention lengths might be another confounding factor. Notably, 78% of the studies included had interventions for an average time of 3 months or less, indicating that changes in physical activity interventions and outcomes could be detected in a short time period, which is highly considered for interventions targeting school- and preschool-age children since they will be present in the school-setting during a school year.

## 5. Strengths and Limitations

This study is the first NMA to examine the effects of different PA interventions on children’s FMSs. Moreover, the included studies represented nine different countries and regions; therefore, the samples could represent children of different cultures and backgrounds. The findings of this review might be generalized to a larger population. In spite of these strengths, our review has some limitations. First, according to the study intervention characteristics, the PA intervention duration or frequencies were not consistent. In addition, limited information regarding PA frequencies or intensity were presented. Second, although this review only retrieved RCTs using TGMD as the sole instrument in assessing FMSs, different versions of this instrument (TGMD-1, TGMD-2, and TGMD-3) might lead to different results. That is, the data accuracy and interpretation might have been compromised across the three versions. Third, the number of included studies employing teacher/parent education interventions is very limited in this review. Implications based on such interventions remain unexplored and need more investigations in the future. Fourth, the authors were unable to test the sensitivity of this review based on intervention durations, given the wide range of PA intervention lengths used in the included studies. Finally, the types of exergaming varied across different studies. Some studies offered a variety of exergames, whereas others only employed certain predetermined exergames, which limited the generalization of specific exergames used in PA interventions.

## 6. Conclusions

In general, children’s FMSs have been improved through different PA interventions. Among them, aerobic interventions seem to be the most effective intervention in enhancing object control skills and overall gross motor skills. As a result, aerobic interventions remain reliable and effective in promoting certain motor skills. Nevertheless, PA intervention program modifications, including intervention length, content, and goals, are warranted to help children and adolescents efficiently develop their motor skills. This NMA also suggests that exergaming is considered the most effective intervention in improving children’s locomotor skills. Therefore, we recommend exergaming as an alternative option for improving children’s motor skills, and consequently, exergaming may be used as a curriculum content in preschools and schools [61,62,63,64]. Additionally, parents and teacher education play an imperative role in children’s FMS development and PA promotion, yet few empirical studies were identified [65]. More research is needed in this area of inquiry.

## Figures and Tables

**Table 1:** Cochrane Risk of Bias Assessment for Randomized Controlled Trials (low, high, unclear).

Study	Random Sequence Generation	Allocation Concealment	Blinding of Participants and Personnel	Blinding of Outcome Assessment	Incomplete Outcome Data Addressed	Selective Reporting
Cliff et al. [44]	low	unclear	low	low	low	low
Barnett et al. [14]	low	low	high	unclear	low	low
Johnstone et al. [15]	low	unclear	high	low	low	low
Jonnson et al. [22]	low	low	low	low	low	low
Lee et al. [45]	high	high	unclear	low	low	low
Chan et al. [23]	low	low	low	low	low	low
McGann et al. [20]	unclear	unclear	high	low	low	low
Vernadakis et al. [21]	low	low	unclear	low	low	low
Sit et al. [32]	unclear	unclear	unclear	low	low	low
Pan et al. [31]	high	high	high	low	low	low
Tzetzis and Kourtessis [46]	unclear	unclear	unclear	low	low	low
Bardid et al. [17]	low	low	unclear	low	low	low
Jones and Riethmuller [18]	low	low	low	low	low	low
Foulkes et al. [19]	low	low	high	low	low	low
Palmer et al. [47]	unclear	unclear	low	low	low	low
Gao et al. [48]	unclear	low	low	low	low	low
Brian et al. [49]	high	high	high	low	low	low
Robinson and Goodway [50]	unclear	low	unclear	low	low	low

Low risk = described adequately within the study; Unclear risk = described somewhat adequately within the study; High risk = was poorly described or not described within the study.

## Data Availability

Not applicable.
